# Asynapsis and meiotic restitution in tomato male meiosis induced by heat stress

**DOI:** 10.3389/fpls.2023.1210092

**Published:** 2023-07-13

**Authors:** Cédric Schindfessel, Nico De Storme, Hoang Khai Trinh, Danny Geelen

**Affiliations:** ^1^ Horticell Lab, Faculty of Bioscience Engineering, Department of Plants and Crops, Ghent University, Ghent, Belgium; ^2^ Institute of Food and Biotechnology, Can Tho University, Can Tho, Vietnam

**Keywords:** meiosis, high temperature, chromosome segregation, cytokinesis, cytomixis, meiotic restitution, clonal gametes

## Abstract

Susceptibility of the reproductive system to temperature fluctuations is a recurrent problem for crop production under a changing climate. The damage is complex as multiple processes in male and female gamete formation are affected, but in general, particularly pollen production is impaired. Here, the impact of short periods of elevated temperature on male meiosis of tomato (*Solanum lycopersicon* L.) is reported. Meiocytes in early stage flower buds exposed to heat stress (>35°C) exhibit impaired homolog synapsis resulting in partial to complete omission of chiasmata formation. In the absence of chiasmata, univalents segregate randomly developing unbalanced tetrads and polyads resulting in aneuploid spores. However, most heat-stressed meiotic buds primarily contain balanced dyads, indicating a propensity to execute meiotic restitution. With most meiocytes exhibiting a complete loss of chiasma formation and concomitantly showing a mitotic-like division, heat stress triggers first division restitution resulting in clonal spores. These findings corroborate with the plasticity of male meiosis under heat and establish a natural route for the induction of sexual polyploidization in plants and the engineering of clonal seed.

## 1 Introduction

Meiosis is a process of two consecutive cell divisions that generates haploid gamete precursor cells developing into pollen and an embryo sac required for fertilization and reproduction. Due to its key role in sexual reproduction, meiosis is highly conserved across eukaryotes, and thereby almost always exhibits the same pattern of a single replication phase followed by two consecutive steps of chromosome segregation. In the first meiotic cell division (MI), the homologous chromosomes separate and in the second division (MII), the two chromatids of each homolog separate, producing four nuclei with a haploid chromosome number from a diploid mother sporophyte (Mercier et al., 2015). Meiosis also reshuffles the parental genomes by homologous recombination, a mechanism that enables the reciprocal exchange of nucleotide strands between parental homologous chromosomes (Wang and Copenhaver, 2018). This process involves the establishment of the synaptonemal complex, a highly structured protein-DNA complex that prepares for the formation of COs which are observed in meiotic chromosome spreads as chiasmata (Mercier et al., 2015). These chiasmata are an important structural element for the coupling of homologous chromosomes in MI and, with each homolog pair forming at least one CO, for imposing a mechanistic framework to secure balanced chromosome segregation in metaphase I. Chromosomes that fail to form a minimum of one CO are subject to random segregation in MI and lead to genomic instabilities in the resulting spores (Zickler and Kleckner, 1998). Micro- and megaspores carrying an imbalanced chromosome set display aneuploidy and usually abort or develop genetic disorders.

Despite a tight self-regulated cell cycle control, the meiotic cell division can exhibit a certain level of plasticity and aberrate from the normal double reductional cell division to yield spores with a non-haploid ploidy level (Enns et al., 2005). One major type of deviation includes all cytological alterations that convert the meiotic cell division into a ‘mitotic-like’ division that results in diploid spores. This defect is referred to as ‘meiotic restitution’ and is caused by either defects in spindle organization, alterations in cell cycle regulation or aberrant cytokinesis (Bretagnolle and Thompson, 1995; De Storme and Geelen, 2013b). From a genetic perspective, meiotic restitution events can be subdivided in two major classes: First Division Restitution (FDR) and Second Division Restitution (SDR). FDR-type restitution mechanisms per definition yield 2n spores that are genetically equivalent to those resulting from the omission of the first meiotic division, and hence produce 2n spores that maintain parental heterozygosity at the centromeric regions. SDR-type restitution mechanisms, on the other hand, generate 2n spores that are genetically equivalent to those resulting from the loss of the second meiotic division, and therefore harbor both chromatids of a single homolog. As a consequence, SDR-type 2n spores are homozygous around the centromeric regions and only maintain parental heterozygosity at the telomeric regions (i.e. due to MI recombination). In plants, studies have shown that shifts in the ambient temperature cause meiotic restitution and the associated production of 2n spores. In rose male meiosis, for example, short periods of heat stress induce ectopic formation of parallel and fused spindles resulting in FDR-type restitution (Pécrix et al., 2011). In *Populus pseudo-simonii* Kitag, heat interferes with the biogenesis of the radial microtubule arrays (RMAs) that mediate cell plate formation at the end of male meiosis II, yielding monads, dyads and triads that contain restituted spores (Wang et al., 2017). In a similar way, cold shock affects the formation of the RMAs in male meiocytes as it results in a higher incidence of diploid pollen formation in *Arabidopsis* (De Storme et al., 2012).

Meiotic plasticity also includes variability in the recombination landscape, i.e. with shifts in the frequency and distribution of CO events. Several studies have shown that the environmental condition can influence both the frequency and the genome-wide positioning of COs, altering patterns of genetic variability in the progeny. In barley, for example, heat stress significantly changes patterns of recombination in male meiosis by shifting chiasmatic events from distal to more proximal chromosome regions (Higgins et al., 2012; Phillips et al., 2015; Schindfessel et al., 2021). Moderate heat stress applied to flowering *Arabidopsis thaliana* leads to an increase in cross overs specifically being formed *via* the type I recombination pathway (Modliszewski and Copenhaver, 2017; Lloyd et al., 2018). Recent cytological studies showed that *Arabidopsis thaliana* male meiosis occasionally exhibits loss of obligate CO formation at elevated temperatures, and thus leads to alterations in MI chromosome segregation and the intrinsic formation of aneuploid spores (De Storme and Geelen, 2020).

Here, we report that high temperature stress interferes with both chiasmata formation and reductional cell division in tomato male meiocytes. Under conditions of heat stress, the homologous chromosomes are not fully synapsed leading to defective recombination and segregation in MI. In parallel, heat also converts the double male meiosis into a single ‘mitotic-like’ division due to aberrations in meiotic spindle organization. The combined defects lead to a *sensu stricto* FDR-type meiotic restitution (De Storme and Geelen, 2013b) and the formation of diploid pollen that are genotypically identical to the mother plant.

## 2 Materials and methods

### 2.1 Plant material and growing conditions

Tomato seeds (*Solanum lycopersicum* cv. Micro Tom) were sown directly into soil and cultivated under controlled conditions of 16h light/8h dark at 20°C. For the various heat treatments, flowering plants were placed in a Panasonic MLR-352H-PE climate chamber under similar conditions, but with elevated temperatures (27°C, 33°C, 35°C or 39°C for 26h or 36h) and/or a different light regime (12h light/12h dark).

### 2.2 Plasmid construction and transformation

Plasmid cloning was performed using the Gateway destination vector pK7m34GW. The promotor Lat52 was cloned in pDONRP4-P1R using primers 5′-GGGGACAACTTTGTATAGAAAAGTTGGTCGACATACTCGACTCAGAAGGTAT-3′ and 5′-GGGGACTGCTTTTTTGTACAAACTTGGTGTCTTGTTTTGATTATA-3′; the CENH3 ORF was cloned into pDONR221 using 5′-GGGGACAAGTTTGTACAAAAAAGCAGGCTCAATGGCGAGAACCAAGCAT-3′ and 5’-GGGGACCACTTTGTACAAGAAAGCTGGGTTCCATGGTCTGCCTTTTCCT-3’. Plants were transformed using the Agrobacterium tumefaciens strain EHA105, as previously described (Swinnen et al., 2022).

### 2.3 Histochemical analysis of male meiosis

Cytological and histochemical analysis of tomato male meiocytes was performed by selecting buds of the appropriate size (2-5mm for meiosis) and squashing isolated anthers in a lactopropionic orcein solution on a microscopy glass slide as described previously (De Storme and Geelen, 2013a).

### 2.4 Male meiotic chromosome spreads

Chromosome spreads of tomato meiocytes were made according to the standard protocol of Ross et al. (1996), with minor modifications (De Storme and Geelen, 2020). Briefly, flower buds of the appropriate size were fixed in 3:1 ethanol:acetic acid fixative for at least 48h. After fixation, these buds were rinsed in two changes of distilled water and two changes of 10mM citrate buffer (pH 4.5). The buds were subsequently digested in a 0.3% cellulase and 0.3% pectolyase enzyme mix for 3h at 37°C. Following the digestion, the buds were squashed on a glass slide, 10µL of 60% acetic acid was added and the suspension was gently stirred on a hot plate (45°C) for 30s. The slide was then flooded with freshly made, ice cold fixative and air dried. Chromosomes were stained with a 2µg/ml DAPI solution in Vectashield antifade medium and mounted with a coverslip.

### 2.5 Immunostaining

Immature flower buds were cut in half and staged using the lactopropionic orcein squash technique. Appropriate buds were fixed for 2 hours in 4% paraformaldehyde in 1X PBS, 0.1% Triton X-100. Isolated anthers were washed twice in 1X PBS and incubated in 0.3% (w/v) enzyme digestion mix (cytohelicase, cellulase RS and pectolyase Y23 [Sigma] in 10 mM citrate buffer) for 3h at 37°C. Following two washes in 1X PBS, single anthers were transferred onto a microscope slide and mechanically disrupted using a blunt needle. The released cells were fixed on the slide by flash freezing in liquid nitrogen and then air dried. The fixed cells were then washed in 1X PBS and incubated in the 0.3% (w/v) enzyme digestion mix for 30 min at 37°C. Next, slides were washed twice with PBS and incubated in PBS with 1% Triton-X for 30 minutes. Following two washes in 1X PBS, squashed meiocytes were labeled with affinity-purified AtASY1 antibody (1:500; Armstrong et al. (2002)) overnight at room temperature. Excess antibody was then removed by four washes in 1X PBS, and labeled with FITC-tagged goat anti-rabbit secondary antibody (1:200; Invitrogen) for 5 hours at 37°C. Finally, the slides were rinsed 5 times with 1X PBS and mounted in Vectashield (Vector laboratories) supplemented with 2 µg/ml DAPI. For cytoskeletal observations using TUBα immunolocalization, a slightly modified protocol was used. Briefly, before fixation, buds were first infiltrated with m-maleimidobenzoyl-N-hydrosuccinimide ester (100 µM in 50 mM PPB and 0.05% [v/v] Triton X-100, pH8) for 30 min under a vacuum to stabilize microtubular (MT) structures. Also, slides were always rinsed in 1X PPB instead of 1X PBS. And finally, the primary TUBα antibody (1:20; Sigma-Aldrich) was detected by FITC labeled goat anti-rat secondary antibody (1:200; Abcam).

### 2.6 Pollen size analysis

Pollen size analysis using the Beckman Multisizer II Coulter counter was performed as previously described (De Storme et al., 2013). Briefly, a single tomato flower at anthesis was vortexed for a couple of seconds in 1ml of Isoton II. Afterwards the flower debris was removed and the suspension was diluted in another 10ml of Isoton II before automatic measurement on the Multisizer II. Data were analyzed using custom R scripts.

### 2.7 Microscopy

Bright-field and fluorescent microscopy images were captured on an Olympus IX81 inverted microscope equipped with an X-Cite 120 LED Boost lamp, using an Olympus XM10 camera and Olympus Cell M software.

### 2.8 Infra-red thermography

Infra-red thermography images of Micro Tom plants inside the climate chamber were made using a FLIR T1030sc camera equipped with a 50µm macro lens. Flower buds were held in place using a metal clamp and a metal needle was placed in the desired focal plane as a high contrast object to allow for better focusing.

### 2.9 Statistical analyzes

Statistical analyses and drawing of graphs was done in R (R Core Team, 2022) using custom scripts. Details of statistical analyses are mentioned in the relevant figure legends or text sections.

## 3 Results

### 3.1 Tomato male meiosis under heat yields aberrant tetrad-stage microspore configurations

**Figure 1:**
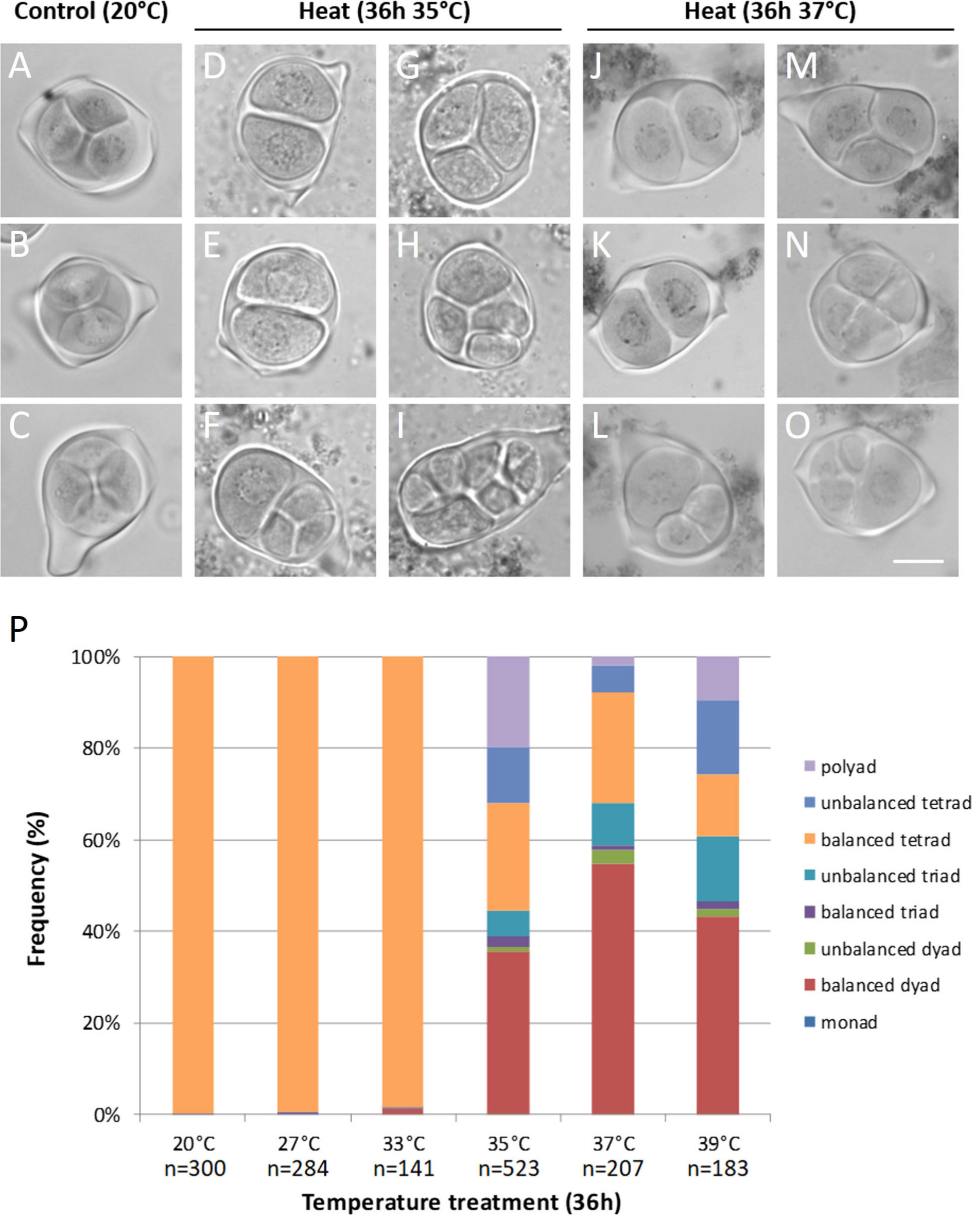
Orcein stained tetrad-stage male meiotic products of Micro Tom grown at a control temperature of 20°C **(A-C)** or after a heat treatment of 36h at 35°C **(D-I)** or 37°C **(J-O)**. Images show representative examples of balanced tetrads **(A-C)**, unbalanced tetrads **(F, N, O)**, balanced triads **(G, M)**, unbalanced triads **(L)**, balanced dyads **(D, E, J, K)** and polyads **(H, I)**. Scale bar = 10µm. **(P)** Quantification of the tetrad-stage configurations observed after various temperature treatments, n represents the number of individual meiocytes counted. At least 3 plants were analyzed per treatment.

Anthers from meiotic flower buds of Micro Tom tomato plants isolated immediately after temperature treatment for 36h at 27°C and 33°C contained predominantly balanced tetrads carrying four equally sized spores, similar as under control (20°C) conditions (**Figures 1A-C, P**). Infrared thermography showed that developing tomato buds take over the surrounding air temperature after about 15min (**Figure S1**), indicating a short lag period. Anthers from flower buds of a similar developmental stage exposed to 35°C, 37°C and 39°C contained a mixture of dyads, triads, tetrads and polyads (**Figures 1D-P**). In the heat stressed anthers, balanced dyads (+/- 40%) were the most abundant configuration followed by normal tetrads (+/- 20%) (**Figure 1P**). The presence of equally sized spores in these meiocytes indicates a balanced segregation of chromosomes and the formation of euploid chromosome sets. A substantial fraction of the meiotic end-products showed a polyad (5 to 20%) or unbalanced tetrad (5 to 15%) configuration, reflecting incorrect MI or MII chromosome segregation (**Figure 1P**). The relative frequency of aberrant microspore configurations did not correlate with the severity of the heat stress and no specific patterns in meiotic cell division defects were discerned across the different treatments. Surprisingly, even the most extreme temperature treatment of 39°C produced balanced tetrads with haploid spores (**Figure 1P**). Overall, the division program of male meiotic cells in tomato anthers is altered at temperatures above 35°C, with the predominant formation of balanced dyads evidencing the induction of meiotic restitution.

### 3.2 Heat stress causes defects in meiotic chromosome dynamics

**Figure 2:**
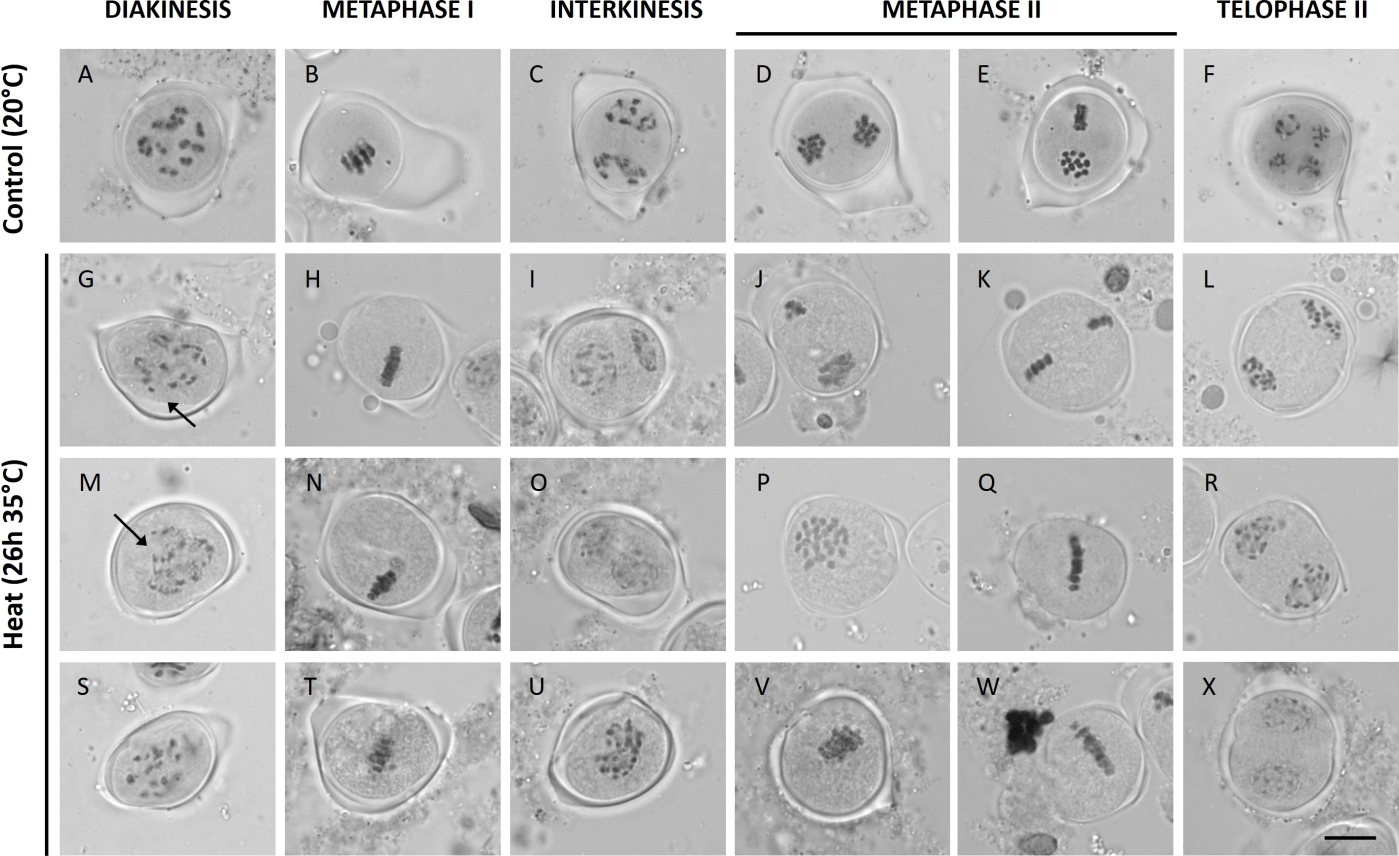
Orcein stained Micro Tom male meiocytes at different stages of development at a control temperature of 20°C **(A-F)**; n = 27 meiocytes) or after a heat treatment of 26h at 35°C **(G-X)**; n = 83 meiocytes). Images include representative examples of diakinesis **(A, G, M, S)**, metaphase I **(B, H, N, T)**, interkinesis **(C, I, O, U)**, metaphase II **(D, E, J, K, P, Q, V, W)** and telophase II **(F, L, R, X)**. Arrows point toward univalent chromosomes. Scale bar = 10µm.

Under control temperatures, Micro Tom male meiocytes display progressive chromosome condensation throughout prophase I forming 12 bivalent chromosome structures that are clearly discerned at diakinesis (**Figure 2A**). In meiosis I, the homologous chromosomes separate to two opposite poles (metaphase I and anaphase I; **Figures 2B, C**), and in meiosis II these two chromosome sets align along perpendicularly oriented planes (**Figures 2D, E**) to eventually separate four sets of 12 chromatids (**Figure 2F**). Under short heat treatment (26h at 35°C), diakinesis-stage meiocytes exhibited an increased number of chromosomal units between 12 and 24, indicating for the presence of ‘unpaired’ chromosomes or univalents (**Figures 2G, M, S**). Despite severe aberrations in bivalent formation, heat-stressed metaphase I meiocytes displayed regular alignment of all chromosomal units at the cytoplasmic midzone (**Figures 2H, N, T**). Later, at interkinesis, two types of chromosomal configurations were discerned: (1) meiocytes with unbalanced chromosome sets at the poles (**Figures 2I, O**) and (2) meiocytes with all chromosomes spatially positioned in the centre of the cytoplasm (**Figure 2U**). Corresponding to these two interkinesis configurations, subsequent alignment and separation of the chromosomes in metaphase II occurs either *via* two spatially separated MII plates with an unequal number of chromosomes (**Figure 2K**), or *via* one single large metaphase II plate that is centrally located within the meiocyte’s cytoplasm (**Figures 2Q, W**). As a result, heat-stressed meiocytes at telophase II displayed two prevailing configurations: namely (1) cells with four or more unbalanced chromosome sets and (2) cells with two enlarged but balanced chromosome sets at the poles (**Figures 2L, R, X**). The occurrence of these two different chromosome segregation patterns in heat-stressed meiocytes corroborates with the distinct types of meiotic end-products formed; namely (1) unbalanced triads, tetrads and polyads and (2) balanced dyads, respectively (**Figure 1**).

Additionally to defects in bivalent formation and MI and MII chromosome segregation, heat-stressed male meiocytes showed events of cytomixis that were undetected in control male meiocytes. About 5% of the heat-stressed meiocytes displayed chromosome strands that are stretched along narrow channels between adjacent meiotic cells, whereas under normal temperatures no cytoplasmic cell-to-cell coupling and intercellular movement of chromosomes is observed (**Figure S2**). Symplasmic channels containing full chromosomes or chromosomal fragments were observed in metaphase I and metaphase II meiocytes, but not at other stages of meiosis.

### 3.3 Heat interferes with homologous recombination and causes loss of obligate chiasma formation

**Figure 3:**
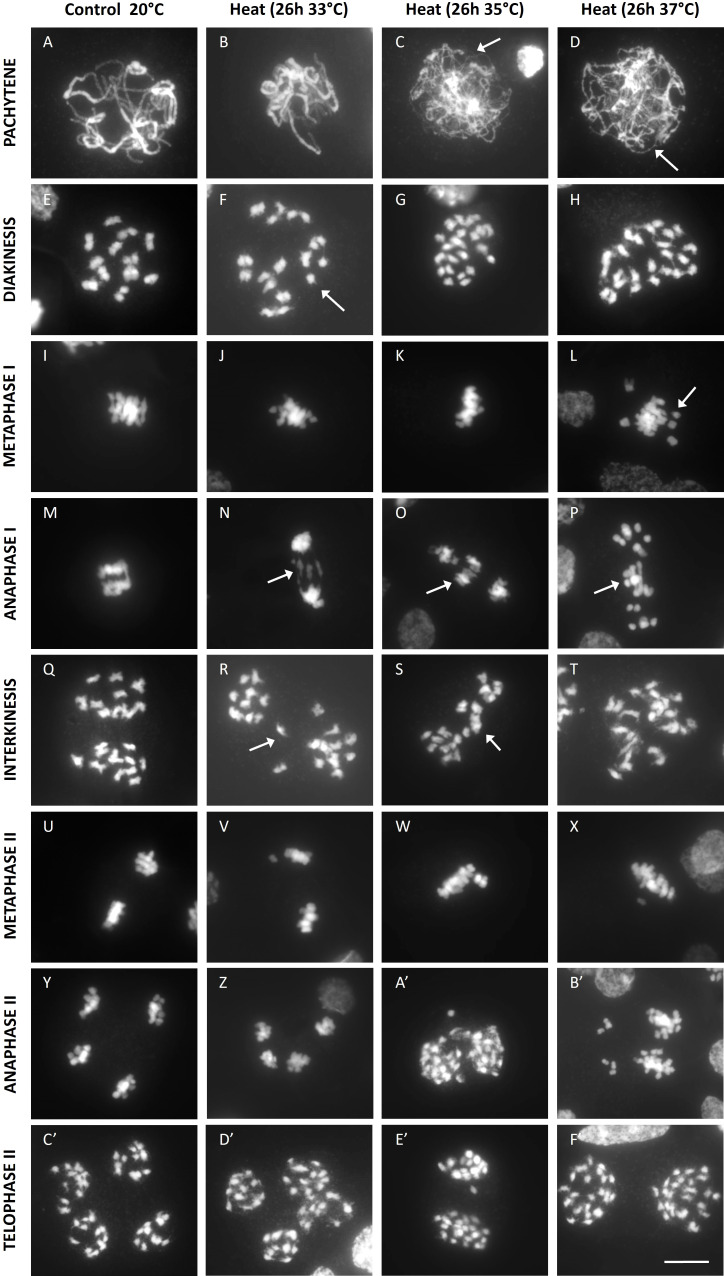
DAPI stained enzyme digested male meiotic chromosome spreads at various stages of Micro Tom grown at a control temperature of 20°C or after a heat treatment of 26h at 33°C, 35°C or 37°C. Images include representative examples of pachytene **(A-D)**, diakinesis **(E-H)**, metaphase I **(I-L)**, anaphase I **(M-P)**, interkinesis **(Q-T)**, metaphase II **(U-X)**, anaphase II **(Y-B')** and telophase II **(C'-F')**. Images were collected from 2 independent treatment experiments for every temperature on different plants. Arrows point toward regions of poor synapsis at pachytene, univalent chromosomes at diakinesis and metaphase I or lagging chromosomes at anaphase I and interkinesis. Scale bar = 10µm.

To further elucidate the cellular mechanism(s) underpinning heat-induced defects in meiotic chromosome dynamics and cell division, male meiotic chromosome spreads from heat-stressed and control plants were compared. A mild temperature elevation to 33°C for 26h did not cause alteration of the pachytene chromosome configuration (**Figure 3B**). At higher temperatures (35°C and 37°C), synapsis was partially impaired or completely abolished (**Figures 3C, D**). At diakinesis, heat-stressed meiocytes formed univalents, and their occurrence was highly temperature-dependent. Meiocytes exposed to 33°C only showed a minor loss of bivalent formation, whereas meiocytes exposed to 35°C and 37°C almost exclusively contained univalents indicating a complete or near-complete loss of chiasma establishment (**Figures 3F–H**). Consistent with the reduced level of CO formation, heat-stressed meiocytes displayed single chromosome units at metaphase I (**Figures 3J–L**) and lagging chromosomes at anaphase I (**Figures 3N–P**). Meiosis I under heat displayed two different outcomes: either (1) two distinct sets of unbalanced chromosomes with occasional laggards (**Figures 3R, S**) or (2) a fully restituted cell with a single set of 24 chromosomes clustered in the cytoplasmic midzone (**Figure 3T**). In meiosis II, all heat-stressed cells showed regular separation of chromatids to opposite poles without additional defects. The pattern by which this occurred depended on the chromosomal configuration at the start of MI. Meiocytes with two unbalanced chromosome sets at interkinesis (**Figure 3W**) were poised to generate unbalanced triads, tetrads or polyads (**Figure 3B’**). In contrast, meiocytes with all chromosomes clustered at interkinesis form one single metaphase II plane (**Figure 3X**) to yield balanced dyads (**Figures 3E, F**).

Overall, depending on the severity of the heat stress, tomato male meiocytes show partial or complete omission of MI homolog synapsis and chiasma establishment, with additional defects in MI chromosome segregation either resulting in imbalanced cell division to yield aneuploid spores or alternatively causing meiotic restitution to form balanced dyads with diploid spores.

### 3.4 Heat induces defects in the morphogenesis and organization of meiotic spindles

**Figure 4:**
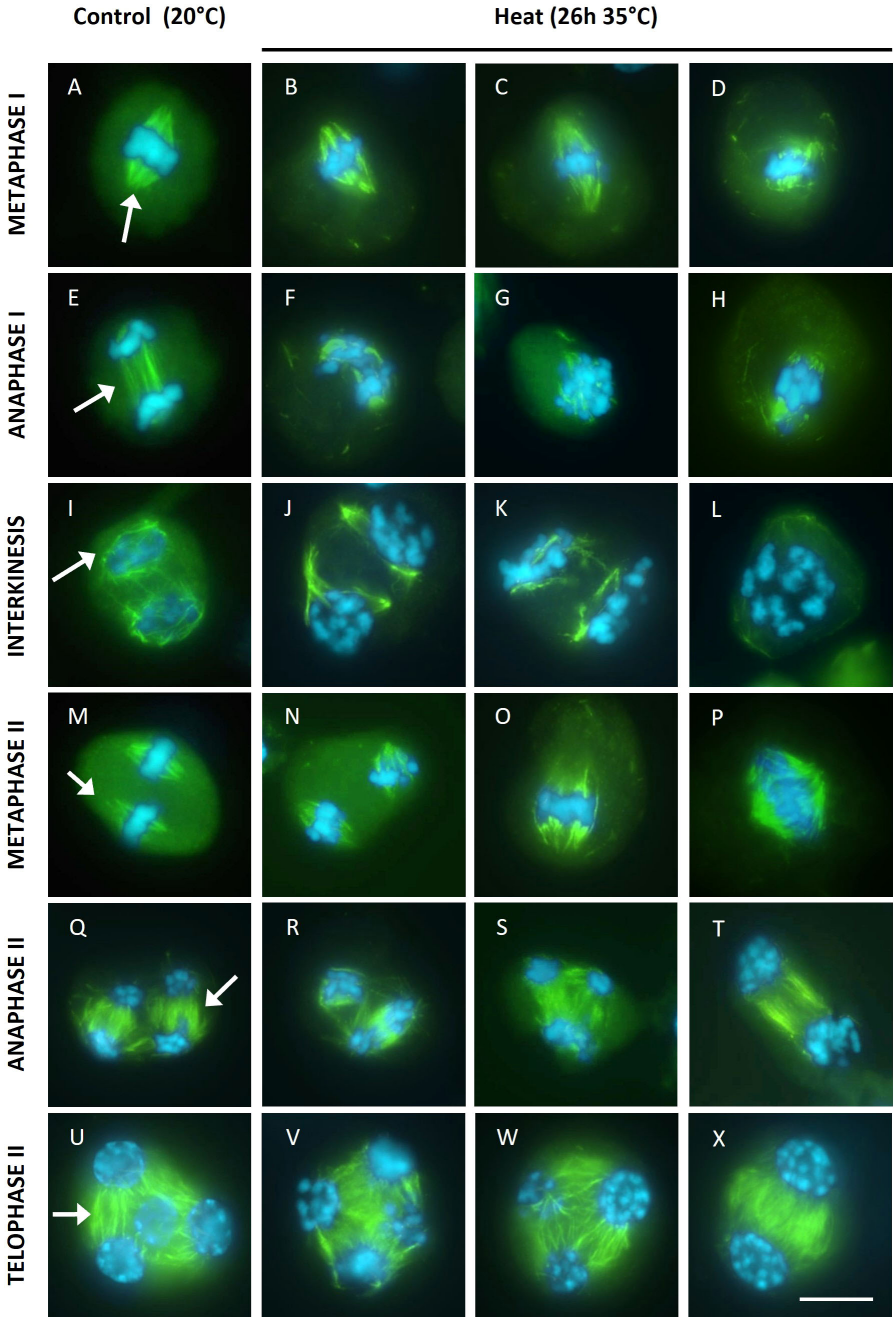
Immuno staining of microtubules (green) on DAPI (blue) stained male meiotic chromosomes at various stages of Micro Tom grown at a control temperature of 20°C or after a heat treatment of 26h at 35°C. Images include representative examples of metaphase I **(A-D)**, anaphase I **(E-H)**, interkinesis **(I-L)**, metaphase II **(M-P)**, anaphase II **(Q-T)** and telophase II **(U-X)**. Images were collected from 4 independent heat treatment experiments on different plants. For the 20°C control, arrows point at well focused spindles at metaphase I and metaphase II, the organised microtubule arrays at anaphase I, anaphase II and telophase II or the ring shaped microtubule array at interkinesis. Scale bar = 10µm.

To assess the putative role of cytoskeletal alterations in chromosome dynamics in heat-stressed tomato flower buds, microtubules (MTs) were immunostained in all meiotic stages. The metaphase I spindles of heat-stressed meiocytes were irregular with unfocused poles and shorter MT bundles (**Figures 4B-D**). In line with these metaphase I spindle defects, anaphase I meiocytes showed two distinct types of aberrant chromosome patterns. Either condensed chromosomes segregated along the disorganized bipolar MT array (**Figure 4F**), or the chromosomes remained gathered at the centre of the cell surrounded by disorganized microtubules (**Figures 4G–H**). Under control temperatures, male meiocytes at interkinesis typically carry two separated chromosome sets that are surrounded by a ring-shaped MT array (**Figure 4I**). In heat-stressed interkinesis meiocytes, chromosomes were either separated in two distinct polar groups or clustered at the cell centre, with both types of chromosome configurations showing a loose association with irregularly organized microtubule arrays (**Figures 4J–L**). In line with this, the metaphase II stage meiocytes also showed two distinct MT patterns. One group of cells displayed two spatially isolated chromosome sets with each set exhibiting a well-structured bipolar spindle, while in the other group all chromosomes were aligned within a single MII spindle (**Figures 4O–P**). At anaphase II, besides regular perpendicularly oriented tetrapolar spindles, also tripolar or fully fused bipolar spindles were observed (**Figures 4T–T**). At telophase II, all heat-stressed meiocytes showed a regular cytoskeletal organization with radial MT arrays emanating from the individual nuclei, however, the number of nuclear domains varied, ranging from two to more than four (**Figures 4V–X**). Meiotic end products with two nuclei consistently showed a balanced chromosome distribution with each nucleus containing roughly the same number of chromosomes (**Figure 4X**), while meiocytes with 3 or more nuclear domains generally displayed an unbalanced chromosome distribution (**Figures 4V–W**).

Overall, heat interferes with the organization of MI spindle structures and MII spindle orientation with cytoskeletal alterations either leading to aberrant chromosome segregation and aneuploidy induction or alternatively conferring meiotic restitution and 2n spore formation due to the ectopic formation of tripolar, parallel and fused spindles in MII.

### 3.5 Heat-induced asynapsis correlates with altered ASY1 dynamics

**Figure 5:**
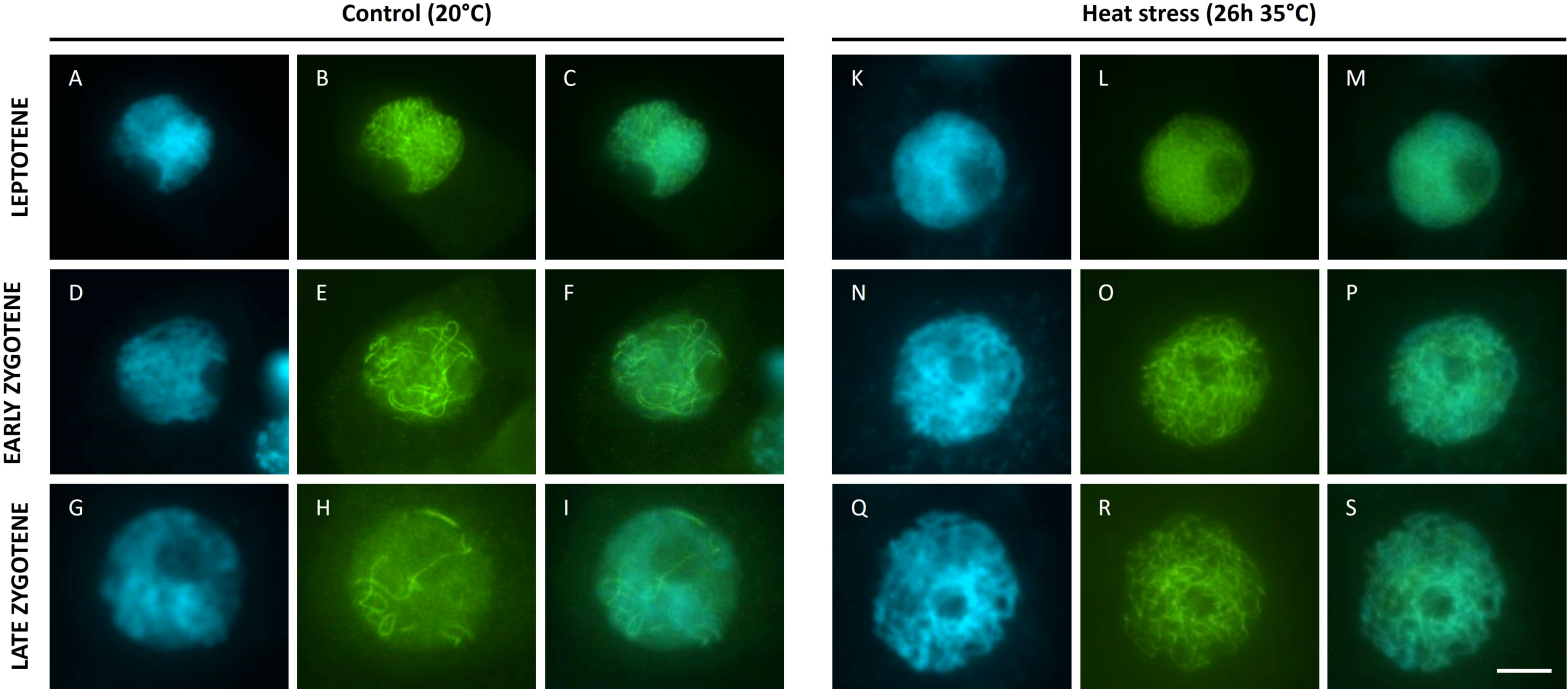
Immuno staining of ASY1 **(B, E, H, L, O, R)** on DAPI **(A, D, G, K, N, Q)** stained male meiotic chromosomes (merged images: **(C, F, I, M, P, S)** at the prophase of Micro Tom grown at a control temperature of 20°C **(A-I)** or after a heat treatment of 26h at 35°C **(K-S)**. Images were collected from 3 independent heat treatment experiments on different plants. Scale bar = 10µm.

The lack of homolog pairing at pachytene upon heat stress suggests that meiotic chromosomes are defective in synapsis. To verify whether heat interferes with the biogenesis of the synaptonemal complex (SC), we attempted to analyse the spatio-temporal dynamics of ASY1 and ZYP1, two key proteins of the SC. ASY1 is a component of the chromosome axis (Armstrong et al., 2002) whereas ZYP1 forms the transverse element between paired homologous chromosomes at pachytene (Higgins et al., 2005). Unfortunately we were unable to visualise the tomato ZYP1 protein using antibodies from *Arabidopsis*, wheat or rice. In contrast, the *Arabidopsis* AtASY1 antibody resulted in chromosome-specific signals in prophase I male meiotic spreads of Micro tom, and thus allows for labelling ASY1 in tomato. Under control temperatures, ASY1 shows a chromosome-specific localization pattern throughout prophase I in accordance with its function as an SC-related axial element (**Figure 5**). Throughout leptotene, the initial ASY1 foci progressively extend to form ‘thread-like’ signals that cover the entire chromosome (**Figures 5A–C**). At early zygotene, ASY1-labelled chromosome strands show regions of paired alignment (**Figures 5D–F**), reflecting sites of physical interaction and pairing between homologous chromosome regions. Throughout zygotene the ASY1 signal progressively disappeared with increasing levels of synapsis (**Figures 5G–I**) to yield meiocytes that completely lack ASY1 at pachytene (**Figure S3** ). Upon heat stress (26h at 35°C), ASY1 foci also appeared on the chromosomes in early stage male meiosis and progressively extended into elongated signals throughout leptotene, similar as in control meiocytes (**Figures 5K–M**). However, the ASY1 labelled strands at leptotene appeared shorter, and at zygotene the difference in length of the ASY1 strands was more pronounced (**Figures 5N–P**). Aligned regions were also absent after heat, and the ASY1 signal was not progressively removed from the chromosomes during prophase I (**Figures 5Q–S**), which is in line with the absence of pairing and synapsis of homologous chromosomes at pachytene, respectively (**Figure 3**).

### 3.6 Heat causes transient male sterility and diploid pollen production in tomato

**Figure 6:**
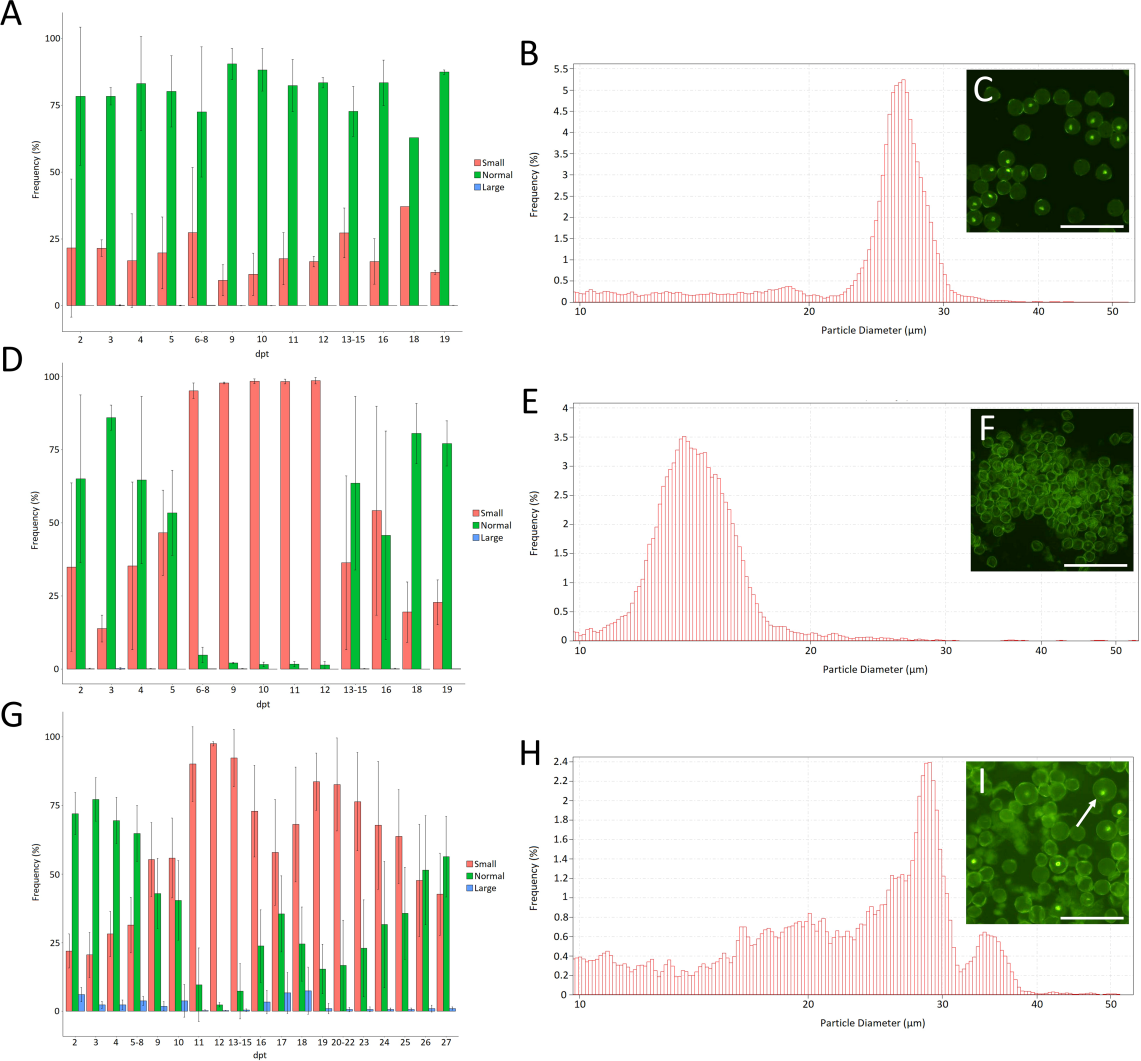
Frequency of small (10µm-20µm), normal (20µm-30µm) and large (30µm-60µm) pollen grains measured by Coulter counter analysis for Micro Tom plants kept at 20°C **(A)** or after a 36h treatment at 35°C **(D)** or 33°C **(G)**. Error bars represent standard errors. Data on every day where gathered from at least 3 individual flowers from 3 individual plants; dpt = days post treatment. **(B)** Example of the pollen size distribution of plants grown at 20°C. **(E)** Example of the pollen size distribution of plants treated for 36h at 35°C on 10dpt. **(H)** Example of the pollen size distribution of plants treated for 36h at 33°C and grown under short day conditions (12h light/12h dark) on 17dpt. The insets **(C, F, I)** show microscopic pictures of the pollen of plants heterozygous for LAT52-CENH3-GFP for the same treatments on the same day after the treatment. The arrow points towards a larger pollen grain. Scale bar = 100µm.

To assess the effects of the proposed heat-induced meiotic defects on the ploidy and genomic stability of resulting microspores and pollen grains, we aimed to generate a pollen-specific centromeric reporter line by transforming tomato plants with a construct expressing a fluorescently tagged CENH3 protein (CENH3-GFP (De Storme et al., 2016)) under the control of the late pollen-specific LAT52 promotor (Twell et al., 1990). This construct specifically labels the vegetative nucleus of mature pollen (**Figure 6C**), allowing for the easy interpretation of the pollen’s DNA content (haploid vs diploid). Heterozygous plants of lines stably expressing this construct were heat treated for 36h at 35°C and their pollen were compared to lines kept at 20°C by means of Coulter counter particle size analysis (De Storme et al., 2013) and fluorescent microscopy.

Under control conditions (20°C) tomato pollen size shows a Gaussian distribution between 20µm-30µm with a peak at around 26µm (**Figures 6A, B**). Upon heat treatment, about 90% of the pollen appear as small particles (<20µm) at 6-12 days following temperature treatment (**Figures 6D-F**). These small particles form a Gaussian distribution around 16µm (**Figure 6E**) and likely represent pollen that aborted during early microspore development. Flowers maturing at 13-16 days following heat shock treatment contain pollen of normal size alongside a large fraction of small particles (<20µm) (**Figure 6D**). Pollen production seemed fully recovered to control levels starting from 19dpt. Altogether these results show that short-term heat stress causes transient defects during early microspore development leading to a temporal period of male sterility in tomato. Similarly, early microspore development was found to be the developmental stage most sensitive to long-term mild heat treatment in a previous study on tomato (Xu et al., 2022).

To test if heat induces the formation of unreduced mature pollen, in accordance with our observations on male meiosis, we reverted to a milder heat treatment of 33°C for 36h on plants grown in short days (12h light/12h dark). In line with the 35°C heat treatment the milder treatment caused severe pollen abortion, represented by the accumulation of smaller particles (<20µm), starting from 4dpt until the end of recording (27dpt). Interestingly, from 16-18dpt we noted a brief window of partial recovery in which slightly more normally sized pollen grains were formed (**Figure 6G**) together with a fraction of enlarged pollen centred around 35µm in size (**Figures 6G-I**). This increase in size (30%) corresponds to unreduced pollen with a ploidy increase from n to 2n (De Storme et al., 2013). In line with this, enlarged pollen expressing CENH3-GFP showed an enlarged vegetative nucleus, indicating an increased ploidy level (**Figure 6I**).

Taking into account the near-complete loss of recombination under heat stress, segregation analysis of the hemizygous CENH3-GFP reporter in larger diploid pollen can be used to determine the extent of FDR- and SDR-type restitution under heat. Quantification of the number of enlarged pollen grains that express the CENH3-GFP protein, and thus harbour the transgenic construct, shows that 98% (n=303) of the larger pollen were fluorescent, indicating that they are predominantly the result of *sensu stricto* FDR (De Storme and Geelen, 2013b).

## 4 Discussion

High temperature stress has pleiotropic effects on both vegetative and generative plant development (Zinn et al., 2010). The reproductive developmental stage is particularly sensitive to heat stress with both cellular and physiological alterations during micro- and mega-gametogenesis reducing fertility (Chen et al., 2016; Rieu et al., 2017). Restituted gametes are of interest to plant breeders that try to create higher level ploidy germplasm (Zhang et al., 2003; Wang et al., 2017), they can play a crucial role in introducing genes from a wild species into a breeding pool with a different ploidy level (Peloquin et al., 1999; Lokker et al., 2005) or can be used to induce apomeiosis (Mieulet et al., 2016; Vernet et al., 2022). Temperature treatment provides an interesting, low-cost, easily applicable and safe alternative to chemical treatment or the genetic engineering of organisms to induce restituted gametes (Dewitte et al., 2012; Lambing et al., 2017). The main pitfall of temperature treatment to tinker with meiosis is the unpredictability of the response of a species or genotype to a certain temperature regime (Bomblies et al., 2015; Lloyd et al., 2018; Coulton et al., 2020; Kuo et al., 2021). Here we show that, in tomato, heat interferes with multiple aspects of male meiosis causing defects in homolog synapsis, the structure and organization of the microtubule cytoskeleton and chromosome segregation that can ultimately lead to the production of clonal gametes.

### 4.1 Heat results in the loss of obligate crossovers

A high temperature induced asynapsis effect has been reported many decades ago in male meiocytes of various organisms (reviewed in Bomblies et al. (2015)) including desert locust (Henderson, 1963), mouse (Nebel and Hackett, 1961) and several plant species, such as *Triticum aestivum* (Bayliss and Riley, 1972) and *Allium ursinum* (Loidl, 1989). In agreement with these earlier reports, heat-stressed tomato meiocytes exhibit impaired synapsis and loss of obligate crossovers resulting in univalent formation in prophase I. A mechanistic link between heat-induced defects in chromosome axis and SC formation and CO establishment has been proposed (Morgan et al., 2017). Heat interferes with the establishment of the key SC protein ZYP1 in both barley (Higgins et al., 2012; Phillips et al., 2015) and *Arabidopsis* (De Storme and Geelen, 2020; Ning et al., 2021). Knock-out mutants of ZYP1, although having a clear impact on CO establishment, only show a minimal loss of obligate crossovers (Capilla-Pérez et al., 2021; France et al., 2021). It thus seems unlikely that an inability to establish the SC is causal for the extreme loss of CO observed in heat stressed tomato. Loss of CO formation is presumably caused by upstream processes that may include double strand-break formation, inter-homolog interactions or double strand-break mediated repair.

### 4.2 Heat induces meiotic restitution

Male meiotic restitution after heat stress has been observed in several plant species including rose (Pécrix et al., 2011), wheat (Rezaei et al., 2010; Omidi et al., 2014), barley (Pao and Li, 1945; Schindfessel et al., 2021), poplar (Wang et al., 2017) and *Arabidopsis* (De Storme and Geelen, 2020). The high temperature conditions applied in our study yielded different types of altered unbalanced meiocytes, i.e., dyads, triads, tetrads and polyads, though balanced dyads and tetrads were the most prevailing meiotic outcomes. Asynaptic mutants defective in e.g. SPO11, DMC1 or ASY1 that produce univalents at the meiotic prophase very rarely show equal segregation of chromosomes with formation of balanced dyads or tetrads (Couteau et al., 1999; Grelon et al., 2001; Dirks et al., 2009). Asynapsis is therefore as such not causing meiotic restitution and diploid gamete formation at the rate (+/- 40%) observed under heat stress.

Fused, parallel or tripolar spindles are often regarded as the main cause of 2n gamete formation under temperature stress (Bretagnolle and Thompson, 1995; Pécrix et al., 2011; De Storme et al., 2012; Wang et al., 2017). Wang et al. (2017) hypothesized that microtubular depolymerisation and incomplete restoration might be an important mechanism underlying 2n gamete formation under heat stress in poplar. Heat-stressed tomato meiocytes showed strong alterations in the microtubular cytoskeleton structure of the metaphase I spindle and subsequent MT configurations. The irregularities were most pronounced at anaphase I with meiosis I spindles that lacked interpolar microtubules. At interkinesis and subsequent MII stages the cytoskeleton organization was strongly affected often forming a single ‘fused’ spindle instead of the regular two perpendicularly arranged spindles and a dyad or triad RMA structure instead of the regular tetrad at telophase II. Since all individual nuclei at the end of MII were separated by a band of microtubules, the increase in ploidy is not a direct consequence of defects in the biogenesis of the RMA, but rather from heat induced damage to MT configurations in meiosis I.

In addition we observed that heat stress causes enhanced cytomixis in tomato, a mechanism that has been proposed to cause unreduced gamete production in plants (Falistocco et al., 1995; Lavia et al., 2011; Mursalimov and Deineko, 2015). The specific timing of cytomixis at metaphase I or II suggests that intercellular chromosome migration between meiocytes under heat stress only occurs when chromosomes are fully condensed and not detained by physical barriers such as the nuclear envelope (at prophase and interkinesis) and the spindle structure (at anaphase I and II). This observation raises the question whether cytomixis is a mechanism that is activated to compensate for the occurrence of a chromosome imbalance in the heat-stressed meiocytes (Mursalimov and Deineko, 2018).

### 4.3 Heat induces a form of apomeiosis

As mentioned above, the loss of obligate crossovers by itself is detrimental for plant fertility. However in combination with the loss of sister chromatid cohesion and skipping the second meiotic division it has previously been used to engineer apomeiosis into *Arabidopsis* and rice (d’Erfurth et al., 2009; Mieulet et al., 2016; Vernet et al., 2022). The heat induced double defect in synapsis and chromosome segregation in tomato hence appears to instigate a form of male apomeiosis that creates clonal spores without genetic engineering. This FDR-type of restitution is not necessarily mimicked in other tomato genotypes or other plant species in which natural genetic variation could have resulted in more (or less) thermotolerant alleles of conserved meiotic genes (Pécrix et al., 2011; Morgan et al., 2017). Indeed, the observations in tomato are in contrast with those in *Arabidopsis* where SDR is more prevalent than FDR under heat stress (De Storme & Geelen, 2020). The restitution in tomato also differs from the impact of heat stress in rose where heat stress induces the formation of parallel spindles in meiosis II, resulting in FDR type restitution but no indications were reported of alterations in crossover formation (Pécrix et al., 2011).

In summary, tomato male meiocytes suffer from multiple defects under heat stress, which lead to alterations in chromosome distribution and ploidy. These findings corroborate with the plasticity of male meiosis to establish a natural route for the induction of sexual polyploidization in plants (Fox et al., 2020; Van de Peer et al., 2021) and offer opportunities for generating clonal spores.

## Data availability statement

The original contributions presented in the study are included in the article/**Supplementary Material**. Further inquiries can be directed to the corresponding author.

## Author contributions

CS, ND and DG designed the experiments. CS, ND and HT performed the experiments. CS and ND analyzed and visualized the data. CS, ND and DG wrote the manuscript. All authors contributed to the article and approved the submitted version.
