# Supplementary material for: Asynapsis and meiotic restitution in tomato male meiosis induced by heat stress
Source: Front Plant Sci. 2023 Jul 13;14:1210092. doi: 10.3389/fpls.2023.1210092 (PMC10373595; doi:10.3389/fpls.2023.1210092)
Supplement: Supplementary file 1 [file DataSheet_1.docx]

Supplementary Material

**Asynapsis and meiotic restitution in tomato male meiosis induced by heat stress**

**Cédric Schindfessel, Nico De Storme, Hoang Khai Trinh and Danny Geelen^*^**

*** Correspondence:** Danny Geelen: Danny.Geelen@ugent.be

# Supplementary Figures


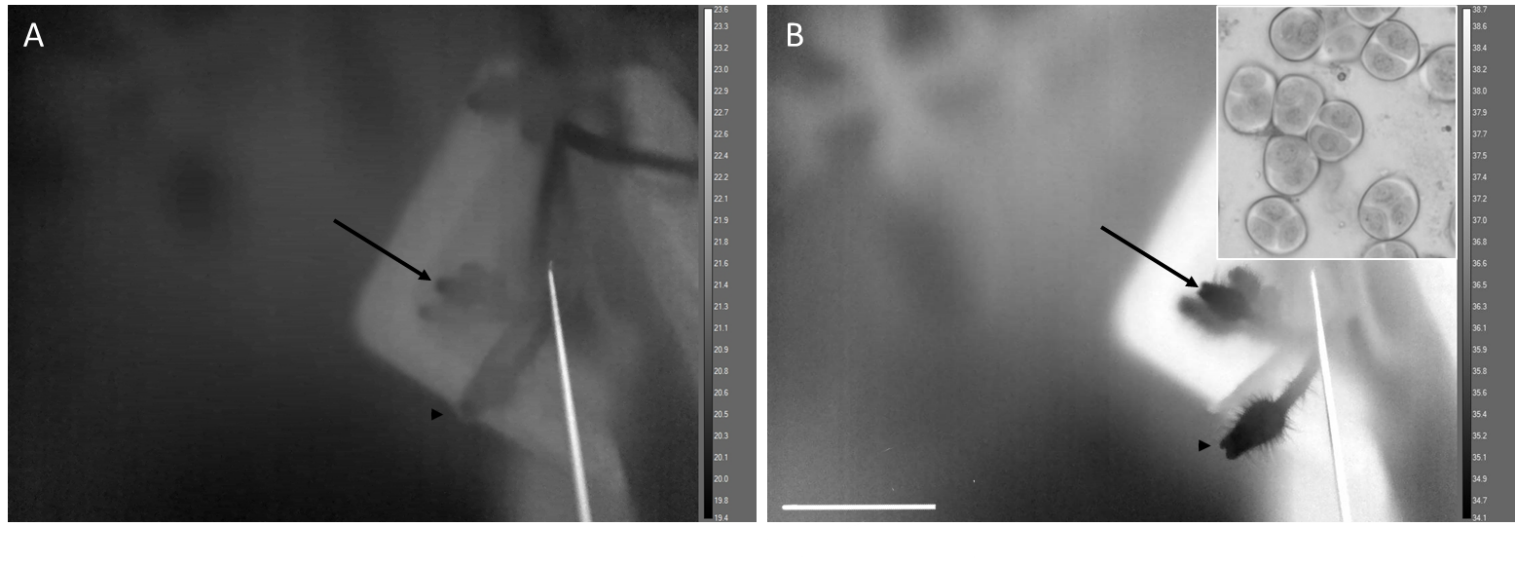


**Supplementary Figure 1.** Infrared thermography images of Micro Tom flowerbuds with ambient temperature set at 20°C (A) or 35°C (B). Images were taken after a stable temperature was reached (approx. 15min) and a dissecting needle placed in the same plane as the buds was used as a high contrast object to focus the image. The average temperature of the buds indicated with the arrow and arrow head are 20.6 and 20.7 respectively in (A) and 35.6, and 35.3°C respectively in (B). The image inset in (B) shows a microscopic image of restituted meiotic products, as found in the bud indicated with the arrow after heat treatment. Scale bar = 10mm.

**
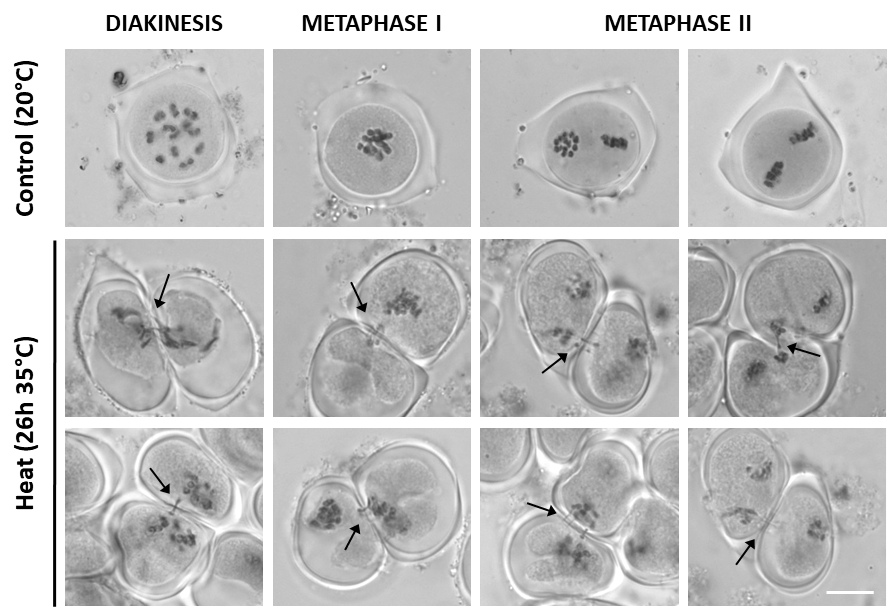
**

**Supplementary Figure 2.** Orcein stained Micro Tom male meiocytes at different stages of development at a control temperature of 20°C or after a heat treatment of 26h at 35°C. Arrows point toward events of cytomixis. Scale bar = 10µm.


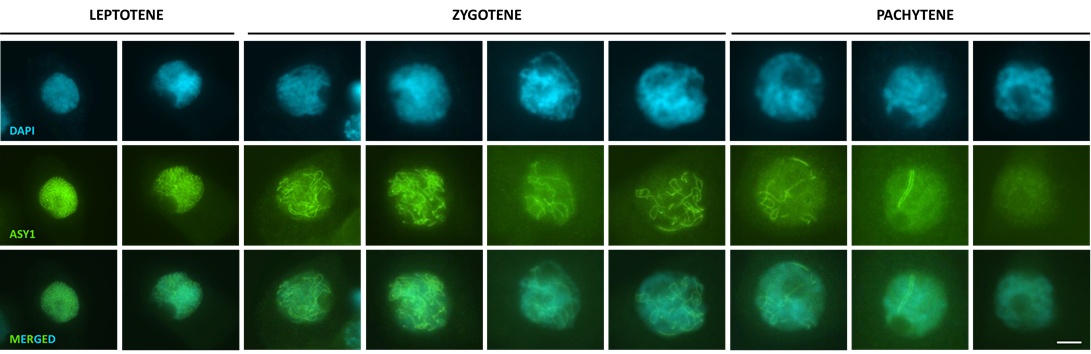


**Supplementary Figure 3.** Immuno staining of ASY1 (green) on DAPI stained male meiotic chromosomes (blue) at different stages of prophase of Micro Tom grown at 20°C. Scale bar = 10µm.
